# Dynamics of post-occlusion water diffusion in stratum corneum

**DOI:** 10.1038/s41598-022-22529-x

**Published:** 2022-10-26

**Authors:** Ivan Argatov, Felix Roosen-Runge, Vitaly Kocherbitov

**Affiliations:** 1 Institut für Mechanik, Technische Universität Berlin, 10623 Berlin, Germany; 2 Faculty of Health and Society, Malmö University, 205 06 Malmö, Sweden; 3 Biofilms-Research Center for Biointerfaces, Malmö University, 205 06 Malmö, Sweden

**Keywords:** Biophysical chemistry, Biophysical chemistry, Drug delivery, Applied mathematics

## Abstract

Diffusion of water through membranes presents a considerable challenge, as the diffusivity often depends on the local concentration of water. One particular example with strong biological relevance is the stratum corneum (SC) as the primary permeability barrier for the skin. A simple alternative for the constant diffusivity model is provided by the Fujita’s two-parameter rational approximation, which captures the experimentally observed fact that the SC diffusion constant for water increases with increasing the water concentration. Based on Fick’s law of diffusion, a one-dimensional concentration-dependent diffusion model is developed and applied for the analysis of both the steady-state transepidermal water loss (TEWL) and the non-steady-state so-called skin surface water loss (SSWL) occurred after removal of an occlusion patch from the SC surface. It is shown that some of the age-related changes in the SSWL can be qualitatively explained by the variation of the dimensionless Fujita concentration-dependence parameter.

## Introduction

The stratum corneum (SC) is the most outer layer of the skin, which is of paramount importance for maintaining the skin’s barrier function integrity. One of the essential functions of the SC is to maintain a healthy level of water loss through the skin, and a detailed understanding of the underlying mechanisms governing the water diffusivity would thus be of high relevance for pharmacy and biomedicine. Typically, the transepidermal water loss (TEWL) as central indicator is evaluated under the conditions of steady state, providing an in vivo assessment of the SC water diffusion. However, this signature misses essential insights into the underlying mechanisms, and should thus be complemented with dynamical methods addressing diffusion outside of the steady state^1^.

One possibility for the in vivo dynamical analysis of water diffusion in SC is provided by the so-called plastic occlusion stress test (POST), which modifies the initial hydration conditions in SC for the evaluation of the skin surface water loss (SSWL). It is suggested that by application of an occlusive patch for a few hours, the occluded SC membrane achieves the fully hydrated state. Therefore, following the removal of the occlusion patch, the water-desorption curve represents the temporary variation of SSWL, which gradually decreases towards the baseline value of TEWL, measured at the same level of ambient humidity.

To date, a number of approaches have been put forward for mathematical modeling of the POST-induced SSWL. A simple biexponential fitting analysis of SSWL was first suggested^2^, based on the analysis of the Fickian diffusion with constant diffusivity. A disposition-decomposition analysis based phenomenological approach was developed^1^ by incorporating epidermal “capacitance” measurements. A two-compartment model, which simulates the SC membrane and the inner skin layers as two biocompartments with different water diffusivities, was introduced^3^ to understand the in vivo cutaneous water balance dynamics. Since the diffusion coefficient of water in SC highly depends on the degree of hydration, the effect of a concentration-dependent diffusivity on the relaxation of perturbed TEWL is expected to be significant^2^.

This study is motivated by a clear problem in interprating experimental evidence based on any model with constant water diffusion coefficient throughout the SC. The SC membrane is known for superior barrier functions, which should be maintained during life in spite of aging. The experimental evidence shows that (**I**) the TEWL does not change with age, (**II**) the initial post-occlusion SSWL is found to significantly increase with age^2^ as well as (**III**) the water content in aged SC is lower than that in young SC^4^. When trying to cope with these facts within the framework of the Fick’s law based model with a constant diffusivity, *D*, we realize that a contradiction arises from combining observations (**I**) and (**II**) with other experimental evidence (**III**) observed for SC. Indeed, it can be shown (see Remark 1 and Section I, SI) that the TEWL and the initial post-occlusion SSWL are respectively proportional to $D/\delta$ and $\sqrt{D}$, where $\delta$ denotes the SC thickness, whereas the water content is simply proportional to $\delta$. That is why, from (**I**) and (**II**), it follows that both *D* and $\delta$ should increase with age, but condition (**III**) implies that $\delta$ decreases with age. This contradiction shows that the constant diffusivity model does not allow to model the age-related alterations in the SC diffusion properties.

A substantial body of experimental evidence reveals the fact that the SC diffusivity is concentration dependent. While clearly more appropriate, a concentration-dependent diffusivity poses essential questions for data interpretation, as the evaluation of experimental signatures becomes in general more complicated. Thus, there is the need to establish a simple but sufficiently flexible functional dependence of the diffusivity *D* on the water concentration, *c*. A good candidate for approximating the function *D*(*c*) is found to be provided by the two-parameter model $D(c)=D_0/(1-\lambda c)$ that was suggested by Fujita^5^ for polymers.

In the present study, we apply the Fick’s law based classical one-dimensional model to water diffusion in SC with a concentration-dependent diffusivity. In terms of the common classification^6^, this model is a transient basic model, which is non-linear due to the dependency of the SC diffusivity on the concentration of the permeating solute. With the aim of obtaining analytical solutions, we introduce the two-parameter Fujita fitting model for describing the SC water diffusivity. An efficient procedure for evaluating the model parameters from the POST-induced variation of SSWL has been developed.

The rest of the paper is organized as follows. In section “Theory”, we develop a one-dimensional diffusion model for a SC membrane based on Fick’s laws and Fujita’s concentration-dependent approximation for the SC diffusivity, by paying a particular attention to the transepidermal water loss (TEWL), water concentration profile, water content, wet thickness of SC, time lag associated with desorption after application of occlusion, and approximately evaluate in explicit form the skin surface water loss (SSWL) as the desorption water flux from a semi-infinite medium. In section “Results”, first, we concretize the main relations for the Fujita approximation-based model and perform its cross-validation by utilizing a data set available in the literature. Then, we apply the developed model for the analysis of post-occlusion SSWL and age-related changes in the SC diffusivity. Finally, we outline a discussion of the obtained results and formulate our conclusions.

## Theory

### Simple fitting approximation for the SC diffusivity

Several different model functions have been proposed to model the water diffusivity as a function of the water concentration. Based on an analysis of experimental data available in the literature, the following formula has been suggested for the diffusivity, *D* ($\mathrm{cm}^2/\mathrm{s}$), of normal human SC^7^:

1 $$\begin{aligned} D\times 10^9 = 0{.}4331-0{.}3765\exp (-9{.}6215 c)+0{.}00006428\exp (12{.}873 c), \end{aligned}$$

which will be referred to as the bi-exponential approximation. Also, based on the analysis performed previously by Stockdale^8^, the following more simple formula has been also suggested^7^:

2 $$\begin{aligned} \frac{D}{D_1}=\frac{0{.}175 c_1}{c_1-c}+0{.}46, \end{aligned}$$

where $$c_1=0{.}835\,\mathrm{g}\,\mathrm{cm}^{-3}$$ and $$D_1=0{.}6\times 10^{-9}\,\mathrm{cm}^2/\mathrm{s}$$.

**Figure 1:**
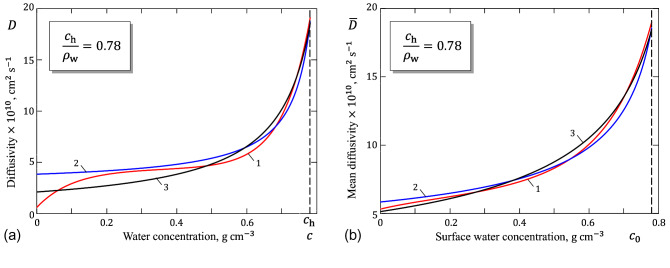
(**a**) Diffusivity of stratum corneum; (**b**) Mean diffusivity of stratum corneum. Curves 1, 2, and 3 correspond to formulas (1), (2), and (3), respectively.

As it was pointed out^7^, formula (2) predicts a steep rise in water diffusivity *D* as the water concentration *c* approaches a limiting value $c_1$, which is taken to be greater than $c_{\mathrm{h}}$, while the latter value was estimated to be $0{.}78\,\mathrm{g}\,\mathrm{cm}^{-3}$ for fully hydrated skin. It is to note here that the second term in formula (1) accounts for a gradual increase in the water diffusivity of dry SC tissue observed in in vitro studies^9^.

As a further simplification over the Stockdale approximation (2), the following two-parameter simple fitting formula has been proposed by Fujita^5^:

3 $$\begin{aligned} D=\frac{D_0}{1-\lambda c}. \end{aligned}$$

The parameters $D_0=2{.}07013\times 10^{-10}\,\mathrm{cm}^2/\mathrm{s}$ and $\lambda =1{.}13887$ of formula (3) are obtained by fitting to formula (1) in the range $0{.}2\le c\le 0{.}78\,\mathrm{g}\,\mathrm{cm}^{-3}$, which covers the common interval of in vivo human skin hydration^7^. The choice of formula (3) was motivated by its utility in describing the growing dependence of the diffusivity *D* on the concentration *c* with a reasonable simply functional form which still allows systematic and physical variations.

The variations of the SC diffusivity as predicted by formulas (1), (2), and (3) are shown in Fig. 1a. An undeniable advantage of formula (3), which was earlier introduced by Fujita^5^ for describing the diffusion of low-molecular-weight substances into high-polymeric solids, is that it allows to derive simple analytical estimates for the dynamic diffusion process.

### Fick’s laws and boundary conditions

To interpret in vivo data on the water diffusion process in stratum corneum (SC), we apply Fick’s second and first laws

4 $$\begin{aligned} \frac{\partial c}{\partial t}=\frac{\partial }{\partial x}\biggl (D(c)\frac{\partial c}{\partial x}\biggr ), \quad J=-D(c)\frac{\partial c}{\partial x} \end{aligned}$$

with a concentration-dependent diffusivity *D*(*c*), where $c=\rho _{\mathrm{w}}\phi _{\mathrm{w}}$ is the water concentration in the SC membrane, $\rho _{\mathrm{w}}$ is the density of water ($1\,\mathrm{g}\,\mathrm{cm}^{-3}$), $\phi _{\mathrm{w}}$ is the water volume fraction, and *J* is the water flux. The SC diffusivity is usually measured in $\mathrm{cm}^2/\mathrm{s}$.

**Figure 2:**
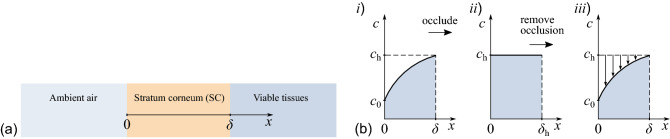
Schematics (**a**) of a SC membrane and (**b**) of the occlusion-induced changes in the water concentration profile across the stratum corneum (after^2^).

For in vivo conditions, we assume that the concentration *c* varies from $c_0$ at the free surface, $x=0$, to the fully hydrated value $c_{\mathrm{h}}$ at a depth where SC merges into the viable tissues (see Fig. 2a)^10^. While the value of $c_{\mathrm{h}}$ is a constant (e.g., $0{.}88\,\mathrm{g}\,\mathrm{cm}^{-3}$, as suggested^10^), the value of $c_0$ depends on the relative humidity of the air. In the steady state conditions, when the concentration *c* does not vary with time, the boundary conditions take the form

5 $$\begin{aligned} c\bigr \vert _{x=0}=c_0, \quad c\bigr \vert _{x=\delta }=c_{\mathrm{h}}, \end{aligned}$$

where $\delta$ is the thickness of SC in vivo.

It is interesting that the SC compartment model^11^ is based on a discrete variant of Fick’s first law (4)$_{2}$.

### Transepidermal water loss (TEWL)

In the steady state, the left-hand side of Eq. (4)$_{1}$ equals zero, and its integration with respect to *x* leads to Eq. (4)$_{2}$ with a constant left-hand side, which will be denoted as $-J_\infty$. In turn, the integration of Eq. (4)$_{2}$ yields the following well-known results^12^:

6 $$\begin{aligned} J_\infty =\frac{1}{\delta }\int _{c_0}^{c_{\mathrm{h}}} D(u)\,\mathrm{d}u =\frac{{\bar{D}}}{\delta }(c_{\mathrm{h}}-c_0), \quad {\bar{D}}=\frac{1}{c_{\mathrm{h}}-c_0}\int _{c_0}^{c_{\mathrm{h}}} D(u)\,\mathrm{d}u. \end{aligned}$$

Here, *u* denotes the integration variable, and ${\bar{D}}$ is the mean diffusion coefficient.

The variation of ${\bar{D}}$ as a function of $c_0$ is illustrated in Fig. 1b for typical variations of the SC diffusivity. Interestingly, the difference between the diffusivity curves diminishes under the operation of averaging. This, in particular, means that experimental errors may have a strong impact on the diffusivity variation *D*(*c*), determined from the measurement of the steady-state water flux, since in such experiments one actually measures the mean diffusivity ${\bar{D}}$.

### Water concentration profile

The water concentration profile, *c*(*x*), is given in the implicit form by the following equation^10^:

7 $$\begin{aligned} \frac{x}{\delta }=\left( \int _{c_0}^{c_{\mathrm{h}}}D(u)\,\mathrm{d}u\right) ^{-1} \int _{c_0}^{c} D(u)\,\mathrm{d}u. \end{aligned}$$

To determine the value *c*(*x*) for a given coordinate *x*, in the general case, Eq. (7) should be solved numerically.

**Figure 3:**
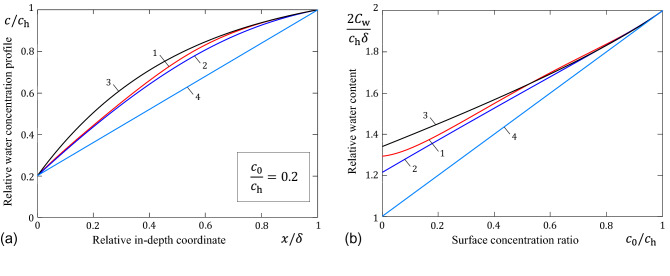
(**a**) Water concentration profile in stratum corneum; (**b**) Water content in stratum corneum. Curves 1, 2, and 3 correspond to formulas (1), (2), and (3), respectively. The straight line (curve 4) represents the constant diffusivity model.

The variation of the relative water concentration profile $c(x)/c_{\mathrm{h}}$ as a function of the relative in-depth coordinate $x/\delta$ is illustrated in Fig. 3a for the typical variations of the SC diffusivity when $c_0/c_{\mathrm{h}}=0{.}2$. Observe that the Fujita approximation (3) overestimates the water concentration profile compared to the bi-exponential approximation (1), though the Fujita parameters were determined by the best fitting to formula (1).

### Water content

Further, by rearranging the steady-state form of Eq. (4)$_{2}$ in the form $c\,\mathrm{d}x=(1/J_\infty )cD(c)\,\mathrm{d}c$, where the constant $J_\infty$ is given by (6), and integrating over the stratum corneum thickness (when $x\in (0,\delta )$ and $c\in (c_0,c_{\mathrm{h}})$), we can evaluate the water content

8 $$\begin{aligned} C_{\mathrm{w}}=\int _0^\delta c\,\mathrm{d}x =\delta \left( \int _{c_0}^{c_{\mathrm{h}}} D(u)\,\mathrm{d}u \right) ^{-1} \int _{c_0}^{c_{\mathrm{h}}} uD(u)\,\mathrm{d}u, \end{aligned}$$

where *c* under the integral sign in the first formula (8) is regarded as a function of the depth *x* according to Eq. (7). It is also to note that the water content is proportional to the wet thickness $\delta$ of the SC membrane.

The variation of the relative water content $2C_{\mathrm{w}}/c_\mathrm{h}\delta$ as a function of the surface concentration ratio $c_0/c_{\mathrm{h}}$ is illustrated in Fig. 3b for typical variations of the SC diffusivity. The dependence on the relative humidity of ambient air enters the right-hand side of formula (8) via the lower integration limit $c_0$.

### Thickness of stratum corneum

Following^10^, we relate the SC thickness to the thickness of the dry SC membrane as

9 $$\begin{aligned} \delta _{\mathrm{d}}=\int _0^\delta \left( 1-\frac{c}{\rho _{\mathrm{w}}}\right) \,\mathrm{d}x, \quad \delta =\delta _{\mathrm{d}}\left( \int _{c_0}^{c_{\mathrm{h}}} \left( 1-\frac{u}{\rho _{\mathrm{w}}}\right) D(u)\,\mathrm{d}u\right) ^{-1} \int _{c_0}^{c_{\mathrm{h}}} D(u)\,\mathrm{d}u, \end{aligned}$$

where Eq. (9)$_{2}$ is derived from Eq. (9)$_{1}$, using Eq. (7).

We note that by the substitution of the second relation (9) into Eq. (8), we can evaluate the water content in the form (see SI, Section V, formula (23)), from where it is readily seen that the in vivo water content of the SC membrane is proportional to the SC dry thickness $\delta _\mathrm{d}$ and is independent of the absolute value of the diffusivity, since the right-hand side of this expression is invariant with respect to the scaling transformation $D\mapsto \Lambda D$ for any positive constant $\Lambda$. However, the value of $C_{\mathrm{w}}$ is sensitive to the form of the functional dependence of the diffusivity *D* on the water concentration *c*.

The SC thickness, $\delta _{\mathrm{h}}$, in the fully hydrated state, when $c_0=c_{\mathrm{h}}$, can be evaluated in the form $\delta _\mathrm{h}=\delta _{\mathrm{d}}(1-c_{\mathrm{h}}/\rho _{\mathrm{w}})^{-1}$. We note that $\delta <\delta _{\mathrm{h}}$ for any $c_0<c_{\mathrm{h}}$.

### Assessment of the SC water diffusivity from the water concentration profile

By rearranging Eq. (4)$_{2}$, we arrive at the following well-known equation^8^:

10 $$\begin{aligned} D(c)=J_\infty \frac{\mathrm{d}x}{\mathrm{d}c}. \end{aligned}$$

The equation above allows to evaluate the function *D*(*c*) in the interval $c\in (c_0,c_{\mathrm{h}})$, provided the water concentration profile is measured in vivo.

### Time lag associated with desorption after application of occlusion

A so-called POST (plastic occlusion stress test) has been proposed^13^ for measuring the water desorption curve after removal of the occlusion. When a water-impermeable plastic patch is applied to the surface of SC, the steady-state water concentration profile changes to reach a uniform distribution, as shown in Fig. 2b. When the occlusive patch is removed, the dynamic desorption process occurs, during which the water concentration profile gradually returns to the steady-state form. This desorption process is characterized by the skin surface water loss (SSWL), which has the same units as TEWL, but is a function of time.

By the definition, we put

11 $$\begin{aligned} J_0(t)=D\bigl (c(x,t)\bigr )\frac{\partial c(x,t)}{\partial x}\biggr \vert _{x=0}. \end{aligned}$$

The initial condition for the third stage of POST is $c\vert _{t=0}=c_{\mathrm{h}}$, $x\in (0,\delta _{\mathrm{h}})$, where $\delta _{\mathrm{h}}$ is the thickness of the fully hydrated SC membrane.

The total water loss (per unit area of the SC free surface) will be given by the integral

12 $$\begin{aligned} Q_0(t)=\int _0^t J_0(\tau )\,\mathrm{d}\tau . \end{aligned}$$

It can be shown^14,15^ that with increasing time the function $Q_0(t)$ approaches the asymptote $Q_{\mathrm{a}}(t)=J_\infty (t+t_{\mathrm{lag}})$, where the time lag $t_\mathrm{lag}$ is given by the following formula:

13 $$\begin{aligned} t_{\mathrm{lag}}=\left( \int _{c_0}^{c_{\mathrm{h}}}D(u)\,\mathrm{d}u \right) ^{-3}\left( \delta ^2 \int _{c_0}^{c_{\mathrm{h}}}D(u)(c_{\mathrm{h}}-u) \int _u^{c_{\mathrm{h}}} D(w)\,\mathrm{d}w\mathrm{d}u\right) . \end{aligned}$$

The time lag is one of the essential parameters accessible in experiments, and expresses the time it would have taken in steady state to loose the excess water loss during the POST protocol.

**Figure 4:**
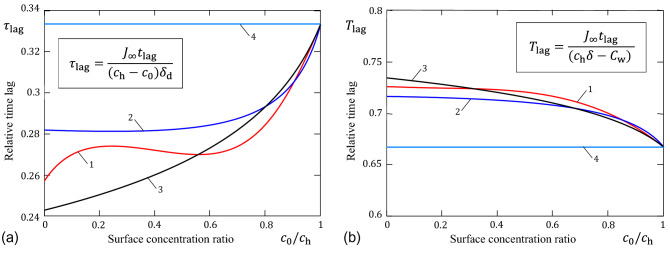
Time lag for stratum corneum relative to the SC dry (**a**) and wet (**b**) bases. Curves 1, 2, and 3 correspond to formulas (1), (2), and (3), respectively. The straight line (curve 4) represents the constant diffusivity model.

To compare the excess water loss $J_\infty t_{\mathrm{lag}}$ during the POST protocol to the water available to diffusion, the relative time lags $\tau _{\mathrm{lag}}=J_\infty t_{\mathrm{lag}}/(c_{\mathrm{h}}-c_0)\delta$ and $T_{\mathrm{lag}}=J_\infty t_{\mathrm{lag}}/(c_{\mathrm{h}}\delta -C_{\mathrm{w}})$ are defined, where $C_{\mathrm{w}}$ is the water content in the steady state. Their variations as functions of the surface concentration ratio $c_0/c_{\mathrm{h}}$ are illustrated in Fig. 4 for the typical variations of the SC diffusivity. It is of interest to note that the variation of $t_{\mathrm{lag}}$ as a function of $c_0$ for the bi-exponential approximation (1) is not monotonic.

We also note that, in view of (12), the following important formula holds true:

14 $$\begin{aligned} t_{\mathrm{lag}}=\int _0^\infty \left( \frac{J_0(t)}{J_\infty }-1\right) \,\mathrm{d}t. \end{aligned}$$

While Eq. (13) allows for the analytical study of the desorption time lag, formula (14) is very useful in the analysis of experimental data. Finally, we note that the value of $t_{\mathrm{lag}}$, as it is readily seen from (13), is positive.

It is interesting to note^15^ that for the diffusion coefficient *D*(*c*) being a strictly-increasing function of the concentration *c*, the desorption time lag is less than the adsorption time lag. This theoretical fact agrees with the simulation results^16^ showing that desorption rates from hydrated SC are faster than hydration rates of dry SC and, apparently, has implications in simulations of the baby diapering scenarios.

#### Remark 1

It is instructive to write out the solutions in the case of constant diffusivity. So, assuming that $D\equiv \mathrm{const}$, Eqs. (6), (7), (8), (9), and (13) yield

15 $$\begin{aligned} c= & {} c_0+(c_{\mathrm{h}}-c_0)\frac{x}{\delta }, \quad J_\infty =(c_{\mathrm{h}}-c_0)\frac{D}{\delta }, \quad t_{\mathrm{lag}}=\frac{\delta ^2}{3D}=\frac{2}{3}\frac{(c_{\mathrm{h}}\delta -C_{\mathrm{w}})}{J_\infty }, \end{aligned}$$

16 $$\begin{aligned} C_{\mathrm{w}}= & {} \frac{(c_{\mathrm{h}}+c_0)}{2}\delta , \quad \delta =\left( 1-\frac{(c_{\mathrm{h}}+c_0)}{2\rho _{\mathrm{w}}}\right) ^{-1} \delta _{\mathrm{d}}. \end{aligned}$$

It interesting to highlight that in this special case, $t_{\mathrm{lag}}$ does not depend on the ratio between $c_0$ and $c_{\mathrm{h}}$.

### Desorption from a semi-infinite medium

In regard to the analysis of a concentration-dependent desorption after application of occlusion up to the so-called breakthrough time^17^, it is of interest to consider the following problem of an semi-infinite interval in contact with a sink. The desorption from a finite medium in the case of constant diffusivity is considered in Supplementary Information (SI) (see Section I).

We have proved (see SI, Section II) that the water flux from a Fujita semi-infinite medium with the initial $c\vert _{t=0}=c_\mathrm{h}$ and boundary $c\vert _{x=0}=c_0$ conditions is given by

17 $$\begin{aligned} J_0(t)=\kappa _1(c_{\mathrm{h}}-c_0)\sqrt{\frac{D_1}{t}},\quad t>0, \end{aligned}$$

where we have introduced the shorthand notation

18 $$\begin{aligned} \kappa _1=\frac{1}{\lambda _1}\sqrt{\frac{2(1-\lambda _1)}{\mu _1}}, \quad D_1=\frac{D_0}{1-\lambda c_0}, \quad \lambda _1=\frac{\lambda (c_{\mathrm{h}}-c_0)}{1-\lambda c_0}, \end{aligned}$$

and the dimensionless parameter $\mu _1$ is defined as the root of the equation

19 $$\begin{aligned} \int _0^1\frac{\mathrm{d}\varphi }{\sqrt{\varphi ^2-2\mu _1\ln \varphi }} =-\frac{1}{2}\ln (1-\lambda _1). \end{aligned}$$

Interestingly, the inverse square root time variation obtained for the water loss (17) is a characteristic feature of the general concentration-dependent desorption from a semi-infinite medium.

Finally, we note that the dimensionless parameter $\kappa _1$, which is defined by formula (18)$_{1}$, depends on the ratio $c_{\mathrm{h}}/c_0$ besides the parameter $\lambda$ that enters the Fujita formula (3).

### Reformulating the boundary conditions in terms of relative humidity

In practice, the surface water concentration $c_0$ cannot be simply controlled in a direct way. Indeed, one can easily control the external relative humidity (RH) and, correspondingly, the surface water activity $a_{\mathrm{w}}^0=\mathrm{RH}/100$%.

The relation between the water volume fraction $\phi _{\mathrm{w}}$ and the water activity $a_{\mathrm{w}}$ is provided by the sorption isotherm $\phi _{\mathrm{w}}=f(a_{\mathrm{w}})$, which is measured in thermodynamic equilibrium under constant temperature. Thus, if $a_{\mathrm{w}}^0$ denotes the water activity at the SC surface, then we will have

20 $$\begin{aligned} c_0=\rho _{\mathrm{w}}f(a_{\mathrm{w}}^0). \end{aligned}$$

Now, by making use of Eq. (20), we can easily recalculate the TEWL (6) as a function of the external relative humidity, provided the sorption isotherm is known. Finally, we note^18^ that the water activity value $a_{\mathrm{w}}^{\mathrm{h}}=0{.}996$ can be assumed on the internal side of SC, which corresponds to 99.6% RH.

## Results

### Main relations of the Fujita approximation-based model

Having presented the basic theoretical relations, we now evaluate the relations for the specific case of the Fujita expression in Eq. (3). By substituting Eq. (3) into Eq. (6)$_{2}$, we obtain the dimensionless form of Fujita’s relation as

21 $$\begin{aligned} \frac{D(c)}{D_0}=\left( 1-\lambda c_{\mathrm{h}}\frac{c}{c_{\mathrm{h}}}\right) ^{-1}, \end{aligned}$$

The mean diffusion coefficient reads

22 $$\begin{aligned} {\bar{D}}=\frac{D_0}{\lambda (c_{\mathrm{h}}-c_0)}\ln \left( \frac{1-\lambda c_0}{1-\lambda c_{\mathrm{h}}}\right) , \end{aligned}$$

so that, in view of (22), formula (6) for the steady-state water loss TEWL can be rewritten as

23 $$\begin{aligned} J_\infty =\frac{D_0}{\lambda \delta }\ln \left( \frac{1-\lambda c_0}{1-\lambda c_{\mathrm{h}}}\right) . \end{aligned}$$

Further, from Eqs. (7) and (8), we arrive at the inverse relation between *c* and *x*, which can be solved for water concentration profile *c*(*x*) as follows:

24 $$\begin{aligned} c=\frac{1}{\lambda }-\left( \frac{1}{\lambda }-c_0\right) \exp \left\{ -\frac{\lambda J_\infty }{D_0}x\right\} . \end{aligned}$$

It is interesting to stress that the water profile is a simple exponential profile, with a spatial decay constant $\lambda J_\infty /D_0$, which, in view of (23), has only one free parameter $\lambda$ besides the geometrical boundary conditions specifying $\delta$, $c_0$ and $c_h$.

The variation of the relative water concentration profile $c(x)/c_{\mathrm{h}}$ as a function of the relative in-depth coordinate $x/\delta$ is illustrated in SI (see Section V, Fig. 6a) for the typical surface concentration ratio $c_0/c_{\mathrm{h}}=0{.}2$. As expected, a small $\lambda$ recovers the constant diffusion result, while a larger $\lambda$ induces a stronger loss of water concentration closer to the surface.

The substitution of Eq. (24) into Eq. (8) yields the water content

25 $$\begin{aligned} C_{\mathrm{w}}=\frac{\delta }{\lambda }+\left( \frac{1}{\lambda }-c_0\right) \frac{D_0}{\lambda J_\infty }\left[ \exp \left\{ -\frac{\lambda J_\infty }{D_0}\delta \right\} -1\right] . \end{aligned}$$

The variation of the relative water content $2C_{\mathrm{w}}/c_\mathrm{h}\delta$ as a function of the surface concentration ratio $c_0/c_{\mathrm{h}}$ is illustrated in SI (see Section V, Fig. 6b) for the typical surface concentration ratio $c_0/c_{\mathrm{h}}=0{.}2$. We remark that even for low humidity, i.e. low $c_0$, a higher $\lambda$ implies that a significant amount of water is retained in the SC compared to the constant diffusivity model.

Now, the substitution of the Fujita expression (3) for the diffusivity into Eq. (9) yields the relative SC thickness

26 $$\begin{aligned} \frac{\delta }{\delta _{\mathrm{d}}}=\left( \frac{c_{\mathrm{h}}-c_0}{\rho _{\mathrm{w}}} +\left( 1-\frac{1}{\lambda \rho _{\mathrm{w}}}\right) \ln \left( \frac{1-\lambda c_0}{1-\lambda c_{\mathrm{h}}}\right) \right) ^{-1}\ln \left( \frac{1-\lambda c_0}{1-\lambda c_{\mathrm{h}}}\right) , \end{aligned}$$

where $\delta _{\mathrm{d}}$ is the SC thickness in the dry state.

From Eqs. (3) and (13), it follows that

27 $$\begin{aligned} t_{\mathrm{lag}}=\frac{\lambda \delta }{J_\infty } \left( \ln \frac{1-\lambda c_0}{1-\lambda c_{\mathrm{h}}} \right) ^{-2} \int _{c_0}^{c_{\mathrm{h}}} \frac{(c_{\mathrm{h}}-u)}{1-\lambda u} \ln \left( \frac{1-\lambda u}{1-\lambda c_{\mathrm{h}}}\right) \,\mathrm{d}u. \end{aligned}$$

We note that $t_{\mathrm{lag}}$ depends on $D_0$ via $J_\infty$.

The variation of the relative time lag $\tau _{\mathrm{lag}}=J_\infty t_{\mathrm{lag}}/(c_{\mathrm{h}}-c_0)\delta$ given by formula (27) as a function of the surface concentration ratio $c_0/c_{\mathrm{h}}$ is illustrated in SI (see Section V, Fig. 7a) for the typical surface concentration ratio $c_0/c_{\mathrm{h}}=0{.}2$. Fig. 7b (see Section V, SI) illustrates the variation of the relative time lag $\tau _{\mathrm{lag}}$ as a function of the concentration-dependence parameter ${\bar{\lambda }}$ for various values of the ratio $c_0/c_{\mathrm{h}}$. Again, the larger the dependence of the diffusivity on the concentration, i.e., the larger $\lambda$, the shorter the lag time, as more water is retained compared to the constant diffusivity model for $\lambda =0$.

Finally, by making use of Eq. (26), we can rewrite formula (23) in the form

28 $$\begin{aligned} J_\infty =\frac{D_0}{\lambda \delta _{\mathrm{d}}} \left( \frac{c_{\mathrm{h}}-c_0}{\rho _{\mathrm{w}}} +\left( 1-\frac{1}{\lambda \rho _{\mathrm{w}}}\right) \ln \left( \frac{1-\lambda c_0}{1-\lambda c_{\mathrm{h}}}\right) \right) . \end{aligned}$$

Thus, the Fujita approximation-based model contains only three governing parameters, namely, $D_0$, $\lambda$, and $\delta _\mathrm{d}$. The limiting case $\lambda \rightarrow 0$ corresponds to the constant diffusivity.

### Cross-validation of the Fujita approximation-based model

To validate the proposed model, we make use of the data set presented by Blank et al.^10^ and employ the inverse analysis to recover the diffusivity data from their TEWL predictions. Since the TEWL variation obtained in^10^ is given as a function of relative humidity, we need to utilize a sorption isotherm in order to recalculate the TEWL versus the surface water concentration.

It can be shown (see SI, Section III, Fig. 3), the equilibrium sorption data for human stratum corneum^19^, which was used in^10^, is well fitted by the GAB (Guggenheim–Anderson–de Boer) model

29 $$\begin{aligned} c_{\mathrm{w}}=\frac{\rho _{\mathrm{w}}\rho _{\mathrm{SC}}V_{\mathrm{w}}}{ \rho _{\mathrm{w}}+\rho _{\mathrm{SC}}V_{\mathrm{w}}}, \quad \frac{V_{\mathrm{w}}}{V_{\mathrm{m}}}=\frac{Ck a_{\mathrm{w}}}{(1-ka_{\mathrm{w}})(1-ka_{\mathrm{w}}+Cka_{\mathrm{w}})} \end{aligned}$$

with the following coefficients^20^: $\rho _{\mathrm{SC}}=1{.}3\,\mathrm{g}\,\mathrm{cm}^{-3}$, $C=4{.}39$, $k=0{.}9901$, and $V_{\mathrm{m}}=0{.}0386$ g water/g dry tissue.

It ought to be emphasized that in the literature there are many studies (on subjects from different age and gender groups) addressing post-occlusion water diffusion in human stratum corneum, which were recently summarized by Saadatmand et al.^21^ and analysed using a numerical skin swelling model^16^ with the biexponential approximation for the SC diffusivity (1) and the GAB sorption isotherm (29). It was concluded that for describing most of the collected SSWL data, in particular, the value of the GAB parameter *k* must be reduced similar to earlier microcalorimetry studies of human SC^22^. In this regard it should be highlighted that the sorption isotherm of SC is known to be influenced by its composition, including the lipid content^23^, so that the GAB parameter *k* is expected to be subject-dependent. In the case under consideration, it has been verified (see SI, Section II, Fig. 2) that the GAB sorption isotherm with the adopted parameters was fitted well to the specific experimental data^19^, which was used in the diffusion analysis^10^.

**Figure 5:**
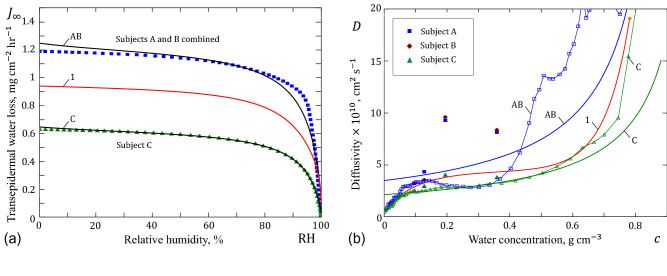
(**a**) Calculated in vivo steady-state water flux in stratum corneum $J_\infty$ as a function of relative humidity; (**b**) Water diffusivity of stratum corneum *D* as a function of water concentration *c*. The symbolized points are adopted from^10^.

It is to emphasize that the TEWL variations (see Fig. 5) were predicted in^10^ based on the experimental data for the SC diffusivity (see Fig. 5b) and the equation

30 $$\begin{aligned} J_\infty =\frac{1}{\delta _{\mathrm{d}}}\int _{c_0}^{c_{\mathrm{h}}} \left( 1-\frac{u}{\rho _{\mathrm{w}}}\right) D(u)\,\mathrm{d}u, \end{aligned}$$

which follows from Eqs. (6) and (9).

However, no analytical approximation for the dependence of *D* on *c* was reported in^10^. Thus, we can regard the TEWL predictions shown in Fig. 5a as our input data for fitting by Eq. (28). Following^10^, we assume that $c_{\mathrm{h}}=0{.}88\,\mathrm{g}\,\mathrm{cm}^{-3}$ and take $\delta _{\mathrm{d}}=10\,\mu \mathrm{m}$ for the SC dry thickness for all subjects (A, B, and C), which experimentally varied from $6\,\mu \mathrm{m}$ to $13\,\mu \mathrm{m}$.

So, by fitting the TEWL variations with Eq. (28), we obtain $D_0=3{.}451\times 10^{-10}\,\mathrm{cm}^2\,\mathrm{s}^{-1}$, $\lambda =1{.}073\,\mathrm{cm}^3\,\mathrm{g}^{-1}$ for subjects A and B, and $D_0=2{.}092\times 10^{-10}\,\mathrm{cm}^2\,\mathrm{s}^{-1}$, $\lambda =0{.}979\,\mathrm{cm}^3\,\mathrm{g}^{-1}$ for subject C. The corresponding curves for the diffusivity *D* are shown in Fig. 5b, where the bi-exponential approximation (1) has been shown as well.

On the other hand, the discrete TEWL variations from Fig. 5a can be used directly to evaluate the SC variable diffusivity coefficients of subjects A, B, and C. In this way, from Eq. (30), it follows that

31 $$\begin{aligned} D=-\delta _{\mathrm{d}}\left( 1-\frac{c}{\rho _{\mathrm{w}}}\right) ^{-1} \frac{\mathrm{d}J_\infty }{\mathrm{d}c}. \end{aligned}$$

Formula (31) allows us to evaluate the diffusivity *D* as a function of the water concentration *c*. It can be used when the TEWL variation $J_\infty (c_0)$ is given in terms of the water concentration $c_0$ at the free surface of the SC membrane.

Further, by utilizing the general sorption isotherm (20), we can transform Eq. (31) as follows:

32 $$\begin{aligned} D=-\frac{\delta _{\mathrm{d}}}{\rho _{\mathrm{w}}\left( 1-f(a_{\mathrm{w}})\right) } \left( \frac{\mathrm{d}f(a_{\mathrm{w}})}{\mathrm{d}a_{\mathrm{w}}} \right) ^{-1}\frac{\mathrm{d}J_\infty }{\mathrm{d}a_{\mathrm{w}}}. \end{aligned}$$

Formula (32) is recommended for the use when the TEWL variation $J_\infty (a_{\mathrm{w}}^0)$ is known in terms of the water activity $a_{\mathrm{w}}^0$ at the SC surface.

The results of the application of Eq. (32) to the discrete TEWL variations shown in Fig. 5a, when the sorption isotherm is described by the GAB model (29) are presented in Fig. 5b. It is of interest to observe that, in contrast to the Fujita approximation (3), the obtained variations of the SC diffusivity exhibit the decreasing trend as the water concentration decreases. However, this feature is captured by the bi-exponential approximation (1). It is to note that the scattering of the diffusivity discrete variations observed in Fig. 5b can be partially explained by the digitalization errors as well as by the numerical errors due to the graphical differentiation employed.

It should be noted that the developed model assumes the Dirichlet-type boundary conditions (5) on both sides of the SC membrane. However, when the surface of skin is exposed to moving air, it is known^16,21^ that the Robin-type boundary condition, which relates the water flux at the skin surface to the difference ($a_{\mathrm{w}}^0-\mathrm{RH}/100$) with a mass transfer coefficient being dependent on the indoor wind velocity, would be more realistic. In this case, in view of Eq. (20), the water activity at the SC outer surface can be evaluated as $a_{\mathrm{w}}^0=f^{-1}(c_0/\rho _{\mathrm{w}})$, where $f^{-1}(\phi _{\mathrm{w}})$ is the inverted sorption isotherm. Correspondingly, the boundary condition at the skin surface will be non-linear and numerical methods will be needed even for solving the steady-state diffusion problem with account for a discontinuity between water activity at the skin surface $a_{\mathrm{w}}^0$ and that in the surrounding air, which is given by $\mathrm{RH}/100$. This effect manifests itself in comparison of Curve 1 from Fig. 5a with the calculated water flux (TEWL) through SC obtained by Li et al.^16^ (see their Fig. 3, where the dry SC thickness is taken to be $9{.}55~\mu \mathrm{m}$). To the best of the authors’ knowledge, there are no published studies on a parametric analysis of this problem that addresses the effect of the boundary layer resistance on TEWL.

### Interpretation of the Fujita parameters

First of all, we observe that both Fujita parameters $D_0$ and $\lambda$ that enter formula (3) are dimensional. In the case of stratum corneum, when the water concentration inside the SC membrane varies between the boundary values $c_0$ and $c_\mathrm{h}$, it makes sense to represent the Fujita approximation (3) in the form

33 $$\begin{aligned} D=D_0\left( 1-{\bar{\lambda }}\frac{c}{c_{\mathrm{h}}}\right) ^{-1}, \quad {\bar{\lambda }}=\lambda c_{\mathrm{h}}. \end{aligned}$$

The relative diffusivity curve $D/D_0$ is shown in Fig. 8a (see Section V, SI) in the logarithmic scale for different values of the concentration-dependence parameter ${\bar{\lambda }}$. Figure 8b (see Section V, SI) illustrates the variation of the relative diffusivity $D(c_0)/D_0$ in the logarithmic scale as a function of the concentration-dependence parameter ${\bar{\lambda }}$ for various values of the ratio $c_0/c_{\mathrm{h}}$. Larger values of $\lambda$ and ${{\bar{\lambda }}}$ induce a larger sensitivity of the diffusion coefficient on the local concentration. As a more practical interpretation, $\lambda$ represents the slope of *D* in the limit of $c=0$, and in addition induces more spread of the diffusivities at larger *c*. We remark that $\lambda$ has to be chosen smaller than $1/c_h$, i.e. ${{\bar{\lambda }}}<1$, to avoid an unphysical divergence of the diffusion coefficient.

We note that in the example considered in section “Cross-validation of the Fujita approximation-based model”, we have ${\bar{\lambda }}=0{.}944$ for subjects A and B, and ${\bar{\lambda }}=0{.}862$ for subject C.

Further, the Fujita parameter $D_0$ has a simple interpretation as the diffusivity at zero concentration, that is $D_0=D(0)$. However, as it was already pointed out before, the Fujita approximation in Eq. (33)$_{1}$ does not describe the variation of the diffusivity for dry SC, and therefore, the value of $D_0$ should not be accepted as characteristic for keratinous tissues^9^.

The diffusivity of SC in the fully hydrated state, which according to Eq. (33)$_{1}$ is predicted to be $D_\mathrm{h}=D_0/(1-{\bar{\lambda }})$, should also be considered with care for characterization of permeabilities of different SC membranes, as the value of $D_{\mathrm{h}}$ is difficult to measure experimentally^24^.

By taking into account the fact that TEWL measurements allow to easily estimate the mean diffusivity, it seems logical to characterize different SC membranes by the mean diffusivity coefficient ${\bar{D}}$ defined by formula (6)$_{2}$ for comparison. However, a complication appears when TEWL tests are performed at different levels of air humidity, as the mean diffusivity ${\bar{D}}$ depends on the water concentration $c_0$ at the free surface. In this case, one can introduce a standard relative humidity $\mathrm{RH}_{\mathrm{s}}$ and, correspondingly, according to the sorption isotherm (20), a standard water concentration at the SC surface as

34 $$\begin{aligned} c_{\mathrm{s}}=\rho _{\mathrm{w}}f\left( \frac{\mathrm{RH}_{\mathrm{s}}}{100}\right) . \end{aligned}$$

Then, in view of (33)$_{2}$, formula (22) yields the Fujita mean diffusivity at standard air humidity

35 $$\begin{aligned} {\bar{D}}_{\mathrm{s}}=\frac{D_0}{{\bar{\lambda }}(1-c_{\mathrm{s}}/c_{\mathrm{h}})} \ln \left( \frac{1-{\bar{\lambda }}c_{\mathrm{s}}/c_{\mathrm{h}}}{1-{\bar{\lambda }}}\right) , \end{aligned}$$

where $c_{\mathrm{s}}$ is fixed according to Eq. (34).

Thus, in the framework of the Fujita approximation (33), the diffusivity properties of SC can be comparatively characterized by the dimensionless concentration-dependence parameter ${\bar{\lambda }}$ and the mean diffusivity ${\bar{D}}_{\mathrm{s}}$, defined by formula (35) for a certain standard level of ambient humidity.

### Analysis of post-occlusion skin surface water loss

From Eq. (17), it follows that the SSWL in some initial time interval $(0,t_1)$ after the occlusion removal varies in such a way that

36 $$\begin{aligned} \sqrt{t}J_0(t)\simeq \kappa _1(c_{\mathrm{h}}-c_0)\sqrt{D_1},\quad t\in (0,t_1). \end{aligned}$$

In view of (18) and (33)$_{2}$, we have

37 $$\begin{aligned} D_0=D_1\left( 1-{\bar{\lambda }}\frac{c_0}{c_{\mathrm{h}}}\right) , \quad \lambda _1=\frac{{\bar{\lambda }}(1-c_0/c_{\mathrm{h}})}{1-{\bar{\lambda }}c_0/c_{\mathrm{h}}}. \end{aligned}$$

The dimensionless factor $\kappa _1$ that enters the right-hand side of relation (36) depends on both the ratio $c_0/c_\mathrm{h}$ of the water concentrations at the boundaries and the dimensionless Fujita concentration-dependence parameter ${\bar{\lambda }}$ via the variable $\lambda _1$ (see Eq. (37)$_{2}$). It can be easily verified that $\lambda _1\in (0,1)$, provided that $c_0<c_{\mathrm{h}}$ and ${\bar{\lambda }}\in (0,1)$. While the physical interpretation is challenging, $\lambda _1$ represents a universal variable, from which realistic values of $\lambda$ can be obtained via rescaling. The variation of the SSWL factor $\kappa _1$ versus $\lambda _1$ is shown in SI (see Fig. 2b). We note that $\kappa _1(0)=1/\sqrt{\pi }=0{.}564\ldots$. Observe that the derivative $\mathrm{d}\kappa _1/\mathrm{d}\lambda _1$ tends to infinity as $\lambda _1$ tends to 1. This means that for the values of ${\bar{\lambda }}$ close to 1, a small error in the determination of ${\bar{\lambda }}$ will lead to a larger error in the evaluation of $\kappa _1$.

Further, we note that as the time variable *t* increases, the SSWL $J_0(t)$ tends to its limit value $J_\infty$ that determines the TEWL. In view of Eq. (33)$_{2}$, Eq. (23) can be represented as

38 $$\begin{aligned} J_\infty =\frac{D_0 c_{\mathrm{h}}}{\delta }\Upsilon _1. \end{aligned}$$

The variation of the TEWL factor $\Upsilon _1$ is shown in SI (see Fig. 4, Section IV).

Finally, by taking into account Eqs. (13) and (33), the time lag can be represented as

39 $$\begin{aligned} t_{\mathrm{lag}}=\frac{\delta ^2}{D_0}\Upsilon _2. \end{aligned}$$

The variation of the time lag factor $\Upsilon _2$ is shown in SI (see Fig. 5, Section IV).

Observe that, generally speaking, formulas (36)–(39) contain three unknown parameters $D_0$, ${\bar{\lambda }}$, and $\delta$, since the surface concentration ratio $c_0/c_{\mathrm{h}}$ is assumed to be specified. For their determination, we have got two equations (38) and (39), and yet one approximate relation, which can be obtained by fitting the approximate formula (36) to the initial SSWL data.

Let us introduce the so-called SSWL intensity factor, $K_0$, as the coefficient of the square-root singularity in the asymptotic approximation

40 $$\begin{aligned} J_0(t)\simeq \frac{K_0}{\sqrt{t}},\quad t\rightarrow 0. \end{aligned}$$

Then, in view of (36), (37), and (40), the approximate relation mentioned above can be written in the form

41 $$\begin{aligned} K_0=c_{\mathrm{h}}\sqrt{D_0}\Upsilon _3. \end{aligned}$$

**Figure 6:**
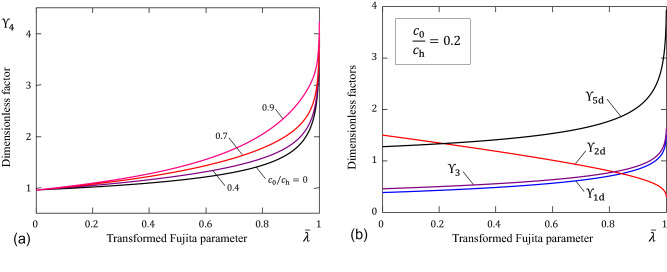
(**a**) Variation of the right-hand side of Eq. (42); (**b**) Variation of the dimensionless factors.

The dimensionless factor $\Upsilon _3$ is given in SI (see formula (18), Section IV) and illustrated in Fig. 6b.

Thus, three equations (38)$_{1}$, (39)$_{1}$, and (41)$_{1}$ can be solved for $D_0$, ${\bar{\lambda }}$, and $\delta$. With this aim, by making use of Eq. (38) and (39), we obtain the relation $D_0=J_\infty ^2 t_\mathrm{lag}c_{\mathrm{h}}^{-2}\Upsilon _1^{-2} \Upsilon _2^{-1}$, from where the wet SC thickness $\delta$ has been excluded. Then, the substitution of the derived expression for $D_0$ into Eq. (41) leads to the equation

42 $$\begin{aligned} \frac{K_0^2}{J_\infty ^2 t_{\mathrm{lag}}}=\Upsilon _4, \end{aligned}$$

where we have introduced the notation $\Upsilon _4=\Upsilon _1^{-2} \Upsilon _2^{-1}\Upsilon _3^2$.

The variation of $\Upsilon _4$ as a function of ${\bar{\lambda }}$ is shown in Fig. 6a. It should be noted that since $\Upsilon _1\rightarrow 1-c_0/c_{\mathrm{h}}$ and $\Upsilon _2\rightarrow 1/3$ as $\lambda _1\rightarrow 0$, we find that $\Upsilon _3\rightarrow 3/\pi$ as ${\bar{\lambda }}\rightarrow 0$ for any value of the ratio $c_0/c_{\mathrm{h}}$.

### Modeling the hypotheses of age-related changes in SC diffusivity

Following^2^, we adopt the following consensus that (**I**) baseline TEWL does not change significantly with increasing age. At the same time, (**II**) initial values of the POST-induced SSWL were found to be significantly greater in old skin than in young^2^. In other words, the SSWL intensity factor $K_0$ increases with age. Moreover, it is also known that (**III**) water content in aged skin is lower than in young^4^, and (**IV**) no young/old differences were noted in the SC thickness^25^. Finally, it as observed^2^ (**V**) relaxation of SSWL, $J_0(t)$, to TEWL, $J_\infty$, is expected to be significantly slower in the aged subjects. In other words, the time lag $t_\mathrm{lag}$ increases with age.

For what follows, it is convenient to represent formulas (26), (38), and (39) in the form

43 $$\begin{aligned} \delta =\delta _{\mathrm{d}}\Upsilon _0, \quad J_\infty =\frac{D_0 c_{\mathrm{h}}}{\delta _{\mathrm{d}}}\Upsilon _{\mathrm{1d}}, \quad t_{\mathrm{lag}}=\frac{\delta _{\mathrm{d}}^2}{D_0}\Upsilon _{\mathrm{2d}}. \end{aligned}$$

For the sake of completeness, we rewrite formula (25) for the water content in two forms (on both a wet and dry basis) as follows:

44 $$\begin{aligned} C_{\mathrm{w}}=c_{\mathrm{h}}\delta \Upsilon _5, \quad C_{\mathrm{w}}=c_{\mathrm{h}}\delta _{\mathrm{d}}\Upsilon _{\mathrm{5d}}. \end{aligned}$$

The dimensionless factors $\Upsilon _0$, $\Upsilon _{\mathrm{1d}}$, $\Upsilon _{\mathrm{2d}}$, $\Upsilon _5$, and $\Upsilon _{\mathrm{5d}}$ are given in SI (Section IV, formulas (19)–(22)).

**Figure 7:**
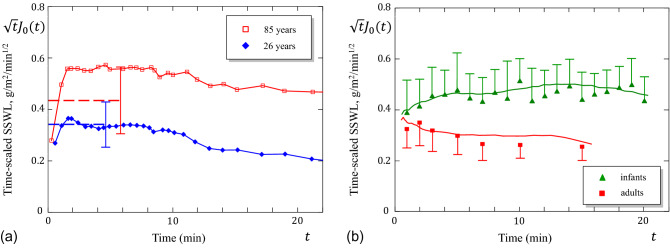
(**a**) Time-scaled variation of the post-occlusion SSWL based on data from Ref.^2^. The dashed lines correspond to the mean values of $J_{30^{\prime \prime }}$ obtained from 26 young (19–42 years) and 18 old (69–85 years) subjects. (**b**) Time-scaled variation of the post-occlusion SSWL based on data from Ref.^26^.

Based on the experimental data obtained by Roskos and Guy^2^, the ratio of the characteristic time for water diffusion across the SC membrane in the old group to that in the young group is found to be $t_{\mathrm{lag}}^{\mathrm{old}}/t_\mathrm{lag}^{\mathrm{young}}=360/175\approx 2.06$ In addition, the ratio $K_0^{\mathrm{old}}/K_0^{\mathrm{young}}$ of the SSWL intensity factors can be estimated from the ratio $J_{30^{\prime \prime }}^\mathrm{old}/J_{30^{\prime \prime }}^{\mathrm{young}}=37/29\approx 1{.}28$ of the SSWL rates at $t=30\,\mathrm{sec}$ post-occlusion (see Fig. 7a).

Based on the experimental data obtained by Zimmerer et al.^26^ we have recalculated the time-scaled variation of the SSWL decay curves, representing the mean of 10 determinations for infants and adults after, respectively, one-hour or two-hour wearing of a diaper or a patch of diaper material. A marked difference is observed between infant and adult skin (see Fig. 7b). It remains a question for further research, and beyond the scope of the present article, to resolve at what age do the water handling properties of skin begin to change.

Therefore, under assumption (**I**) the quantity $K_0^2/J_\infty ^2 t_{\mathrm{lag}}$ on the left-hand side of Eq. (42) markedly decreases with age. Thus, since the quantity $\Upsilon _4$ on the right-hand side of Eq. (42) depends only on one diffusion parameter, we conclude that, in the framework of the developed model (see Fig. 6), the dimensionless Fujita concentration-dependence parameter ${\bar{\lambda }}$ should *decrease* with age.

To verify this hypothesis, we consider the variation of the water content with age, as it follows from Eqs. (44), this quantity is essentially independent of the dimensional diffusivity parameter $D_0$. From Fig. 6b, it is seen that the water content factor on dry basis $\Upsilon _{\mathrm{5d}}$ decreases with decreasing ${\bar{\lambda }}$, which is in agreement with assumption (**III**) under assumption (**IV**).

Further, according to assumption (**V**), the time lag $t_\mathrm{lag}$ increases with age, and this trend is mirrored in the variation of the time lag factor on dry basis $\Upsilon _{\mathrm{2d}}$ when the Fujita parameter ${\bar{\lambda }}$ decreases (see Fig. 6b).

Finally, the effect of the diffusivity parameter $D_0$ manifests itself in both SSWL and TEWL (see Eqs. (40), (41), and (43)). We observe that according to assumption (**II**) the SSWL intensity factor $K_0$ increases with age by about 30%^2^, whereas the effect of decreasing ${\bar{\lambda }}$ on $K_0$ is opposite (see the variation of the SSWL factor $\Upsilon _3$ in Fig. 6b). Hence, the SC diffusivity parameter $D_0$ should increase with age. When regarding assumption (**I**), the effect of increasing $D_0$ on TEWL is compensated by the TEWL factor on dry basis $\Upsilon _{\mathrm{1d}}$, which should decrease with age (see Fig. 6b).

## Discussion and conclusions

Let us recall that the developed model contains a number of parameters, namely, the two Fujita diffusivity constants $D_0$ and ${\bar{\lambda }}$, the dry SC thickness $\delta _{\mathrm{d}}$, and the water concentration $c_{\mathrm{h}}$ of fully hydrated SC. In addition, the water concentration at the free surface $c_0$ is a controlling parameter, which varies in response to the change of the water activity at the SC free surface $a_{\mathrm{w}}^0$ according to the sorption isotherm (20), whose functional dependence may depend on a couple of fitting constants (see, e.g., the GAB model (29)).

In our numerical analysis, we assumed that $c_{\mathrm{h}}=0{.}88$^10^, but there would be only small changes in Fig. 6, if one takes $c_{\mathrm{h}}=0{.}78$^7^. The effect of the sorption isotherm variation^27^ will be seen in the dependence of TEWL as a function of the external relative humidity (see Fig. 5). Therefore, if experimental data for TEWL are reported in terms of relative humidity, then for correct evaluation of the SC diffusivity versus the water concentration, the corresponding sorption isotherm should be reported as well.

**Figure 8:**
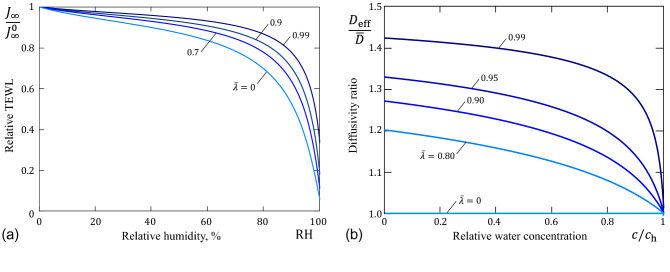
(**a**) Variation of the ratio of the transepidermal water flux $J_\infty$ to the maximum value $J_\infty ^0=J_\infty \vert _{a_\mathrm{w}^0=0}$ versus the environmental RH; (**b**) Ratio of the effective diffusivity $D_{\mathrm{eff}}$ to the mean diffusivity ${\bar{D}}$.

As it was previously observed^10^, the transepidermal water flux keeps a nearly constant value as the environmental RH varies from 0% to 80%. It is of interest to consider the effect of the dimensionless Fujita diffusivity parameter ${\bar{\lambda }}$ on the variation of TEWL. Fig. 8a shows that the ability of SC to maintain the TEWL that does not vary much in this range of RH slightly depends on the value of ${\bar{\lambda }}$. So, in view of age-related variations of ${\bar{\lambda }}$, this SC barrier function may be expected to weaken with age. However, it should be noted that the age effect on the sorption isotherm has been neglected, as the relative TEWL curves in Fig. 8a are drawn for the same GAB isotherm (29).

It should be noted that though the GAB isotherm (29) shows good fitting of experimental results (see SI, Section III, Fig. 3), there are difficulties in interpreting the GAB parameters from a thermodynamic point of view^20^. Moreover, in the context of the present theoretical framework it remains an open question to be addressed in future studies whether the sorption isotherm of stratum corneum changes significantly with age.

It is of interest to compare the mean diffusivity ${\bar{D}}$, which is measured in the steady state diffusion experiments, with the so-called effective diffusivity, $D_{\mathrm{eff}}$, which can be measured from the initial POST-induced SSWL, provided the data is analyzed using the constant diffusivity model (i.e., by fitting the initial part of the relative post-occlusion SSWL $J_0(t)/(c_\mathrm{h}-c_0)$ to the asymptotic approximation $\sqrt{D_{\mathrm{eff}}/\pi t}$). It is to emphasize that the mean diffusivity ${\bar{D}}$ is determined by formula (6)$_{2}$ that also follows from the constant diffusivity model. Thus, if the membrane diffusivity follows the Fujita model (3), then, in view if (17), we have $D_{\mathrm{eff}}=\pi \kappa _1^2 D_1$, where $\kappa _1$ and $D_1$ are given by Eqs. (18)$_{1}$ and (18)$_{2}$, respectively. The variation of the ratio of $D_{\mathrm{eff}}$ to ${\bar{D}}$, which is given by Eq. (22), is shown in Fig. 8b, from where it is seen that the difference between the steady-state and dynamic methods for assessing the concentration-dependent diffusivity may be significant.

Finally, let us discuss some possible ways of generalization of the model that can be dealt with analytical mathematical tools. In particular, following Sütterlin et al.^28^, the flow of calcium, by directed transport coupled to the water flow, can be introduced into the one-dimensional model. On the other hand, there is experimental evidence that the SC membrane hydrates in a non-uniform manner^29^. However, to account for the effect of the SC morphology, one needs to apply at least a two-dimensional brick-and-mortar geometry modeling framework^6^.

To conclude, the developed mathematical modeling framework has a high potential for analyzing the effect of the dependence of the SC diffusivity on the water concentration, which may change in response to a number of factors, including age-related physiologic changes. We remark that the developed solution of the Fujita model is not only relevant for the biological SC, but can as well be used to describe and predict diffusivities in synthetic membranes with similar overall functionality of water diffusion on local concentration.

## Supplementary Information

Supplementary Information.

## Data Availability

The datasets used and/or analysed during the current study available from the corresponding author on reasonable request.
