# Supplementary material for: Dynamics of post-occlusion water diffusion in stratum corneum
Source: Sci Rep. 2022 Oct 26;12:17957. doi: 10.1038/s41598-022-22529-x (PMC9606019; doi:10.1038/s41598-022-22529-x)
Supplement: Supplementary file 1 — Supplementary Information. [file 41598_2022_22529_MOESM1_ESM.pdf]

Supporting Information

Dynamics of post-occlusion water diffusion in stratum corneum

Ivan Argatov
Institut für Mechanik, Technische Universität Berlin, 10623 Berlin, Germany
Faculty of Health and Society, Malmö University, SE-205 06 Malmö, Sweden

Felix Roosen-Runge and Vitaly Kocherbitov*
Faculty of Health and Society, Malmö University, SE-205 06 Malmö, Sweden
Biofilms – Research Center for Biointerfaces, Malmö University, SE-205 06 Malmö, Sweden

In this Supporting Information we present additional details for the SC concentration-dependent diffusion model.

I. DESORPTION FROM A FINITE MEDIUM

In the case of constant diffusivity, it can be shown [1] that the exact solution for the SSWL in the desorption problem, when Fick's second law is applied on the finite interval $x \in (0, \delta)$, can be represented as

$$J_0(t) = \frac{(c_{\rm h} - c_0)D}{\delta}\left(1 + 2\sum_{n=1}^{\infty} \exp\left(-\frac{n^2\pi^2 Dt}{\delta^2}\right)\right), \tag{1}$$

or, which yields the same numerical results, as

$$J_0(t) = (c_{\rm h} - c_0)\sqrt{\frac{D}{\pi t}}\left(1 + 2\sum_{n=1}^{\infty} \exp\left(-\frac{n^2\delta^2}{Dt}\right)\right). \tag{2}$$

In view of the equation

$$J_\infty = (c_{\rm h} - c_0)\frac{D}{\delta},$$

formulas (1) and (2) can be rewritten in the form

$$\frac{J_0(\tau)}{J_\infty} = 1 + 2\sum_{n=1}^{\infty} \exp\left(-\frac{n^2\pi^2\tau}{3}\right) \tag{3}$$

and

$$\frac{J_0(\tau)}{J_\infty} = \sqrt{\frac{3}{\pi\tau}}\left(1 + 2\sum_{n=1}^{\infty} \exp\left(-\frac{3n^2}{\tau}\right)\right), \tag{4}$$

where we have introduced the notation

$$\tau = \frac{3D}{\delta^2}t. \tag{5}$$

We note that formulas (2) and (4) are efficient at the initial interval of desorption, whereas formulas (1) and (3) are better to apply for longer times.

Fig. 1 shows the variation of the relative SSWL J_0/J_∞ as a function of the dimensionless time variable τ. We note that τ has been introduced using the time lag (see also Eq. (5)). Thus, from Fig. 1, it is seen that the SSWL intensity factor $\sqrt{\tau}J_0(\tau)$ keeps a constant value in the half-time lag interval. It is also to note that $\sqrt{\tau}J_0(\tau)$ grows as $J_\infty\sqrt{\tau}$ as τ tends to infinity (this range of τ is not shown in Fig. 1).

---

*Corresponding author: vitaly.kocherbitov@mau.se

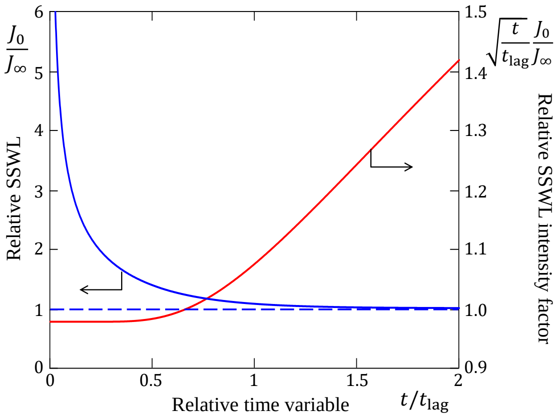

FIG. 1: Desorption from a finite medium in the case of constant diffusivity.

II. DESORPTION FROM A SEMI-INFINITE MEDIUM

In regard to the analysis of a concentration-dependent desorption after application of occlusion up to the so-called breakthrough time [2], it is of interest to consider the following problem of an semi-infinite interval in contact with a sink:

$$\frac{\partial c}{\partial t} = \frac{\partial}{\partial x}\left(D(c)\frac{\partial c}{\partial x}\right), \quad x > 0, \quad t > 0, \tag{6}$$

$$c\big|_{t=0} = c_{\text{h}}, \quad c\big|_{x=0} = c_0. \tag{7}$$

When the diffusion coefficient varies with concentration according to formula (**??**), the exact analytical solution to the transient diffusion problem (6), (7) was first obtained by Fujita [3], though in the case of sorption only.

Let us introduce the change of variable

$$c = c_0 + (c_{\text{h}} - c_0)\theta, \tag{8}$$

where $0 \leq \theta \leq 1$.

The substitution of (8) into Fujita's approximation yields

$$D = \frac{D_1}{1 - \lambda_1 \theta}, \tag{9}$$

where

$$D_1 = \frac{D_0}{1 - \lambda c_0}, \quad \lambda_1 = \frac{\lambda(c_{\text{h}} - c_0)}{1 - \lambda c_0}, \tag{10}$$

and, respectively, Eqs. (6), (7) transform as

$$\frac{\partial \theta}{\partial t} = \frac{\partial}{\partial x}\left(D(\theta)\frac{\partial \theta}{\partial x}\right), \quad x > 0, \quad t > 0, \tag{11}$$

$$\theta\big|_{t=0} = 1, \quad \theta\big|_{x=0} = 0, \tag{12}$$

where $D(\theta)$ is given by formula (9).

By the application of the Boltzmann transformation to dimensionless and rescaled units

$$y = \frac{x}{2\sqrt{D_1 t}}, \tag{13}$$

the transformed problem (11), (12) further reduces to

$$-2y\frac{\mathrm{d}\theta}{\mathrm{d}y} = \frac{\mathrm{d}}{\mathrm{d}y}\left(\frac{D(\theta)}{D(0)}\frac{\mathrm{d}\theta}{\mathrm{d}y}\right), \quad 0 < y < \infty, \tag{14}$$

$$\lim_{y\to\infty}\theta = 1, \quad \lim_{y\to 0}\theta = 0. \tag{15}$$

In the case (9), the boundary-value problem (14), (15) has the following parametric solution [4]:

$$\theta = \frac{r_1}{r_1 - 1}\Big(1 - \exp\Big\{2\big[\mathcal{J}(\phi) - \mathcal{J}(1)\big]\Big\}\Big), \tag{16}$$

$$y = \sqrt{\frac{r_1}{2\mu_1}}\big(j(\phi) - \phi\big)\exp\big\{\mathcal{J}(1) - \mathcal{J}(\phi)\big\}. \tag{17}$$

Here we have introduced the auxiliary notation

$$r_1 = \frac{1}{1-\lambda_1}, \quad j(\phi) = \sqrt{\phi^2 - 2\mu_1 \ln\phi}, \quad \mathcal{J}(\phi) = \int_0^{\phi}\frac{d\varphi}{j(\varphi)}. \tag{18}$$

Moreover, the dimensionless parameter μ_1 is defined as the root of the equation $2\mathcal{J}(1) = \ln r_1$, that is

$$\int_0^1 \frac{d\varphi}{\sqrt{\varphi^2 - 2\mu_1\ln\varphi}} = -\frac{1}{2}\ln(1-\lambda_1). \tag{19}$$

The variation of the function $\theta(y)$ is shown in Fig. 2a for different values of the parameter λ_1.
Observe that $\phi \in (0,1)$, and $y \to 0$ as $\phi \to 1$, so that we have

$$J_0(t) = \frac{D(\theta)}{2\sqrt{D_1 t}}\frac{d\theta}{dy}\bigg|_{y=0} = \frac{D(\theta)}{2\sqrt{D_1 t}}\frac{d\theta/d\phi}{dy/d\phi}\bigg|_{\phi=1}. \tag{20}$$

Then, the substitution of (16), (17) into Eq. (20), in view of (18) and (19), yields

$$J_0(t) = \kappa_1(c_h - c_0)\sqrt{\frac{D_1}{t}}, \quad t > 0, \tag{21}$$

where we have introduced the shorthand notation

$$\kappa_1 = \frac{1}{r_1 - 1}\sqrt{\frac{2r_1}{\mu_1}}. \tag{22}$$

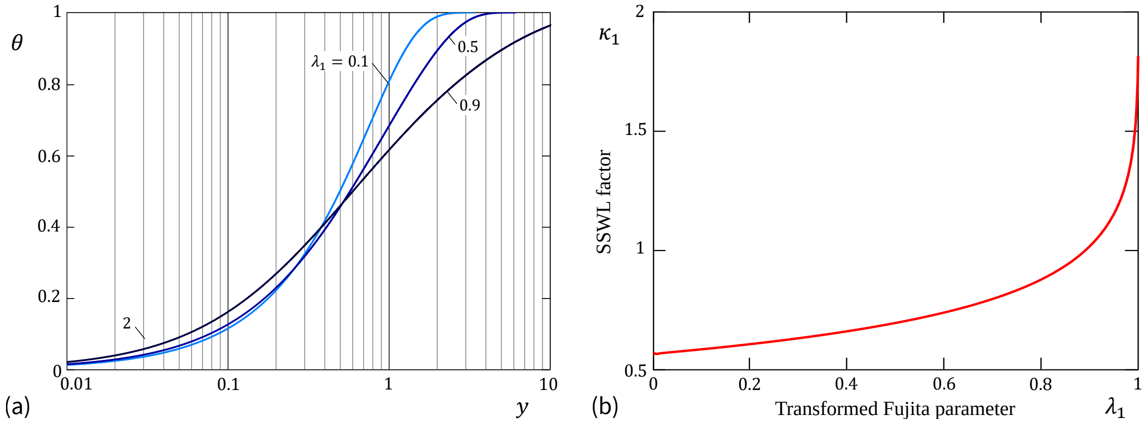

FIG. 2: a) Variation of the function $\theta(y)$ parametrically defined by Eqs. (16) and (17); b) Skin surface water loss factor (22).

III. SORPTION ISOTHERM FOR STRATUM CORNEUM

Fig. 3 shows the fit of the GAB (Guggenheim—Anderson–de Boer) model to the equilibrium sorption data for human stratum corneum [5], which was used in Ref. [6].

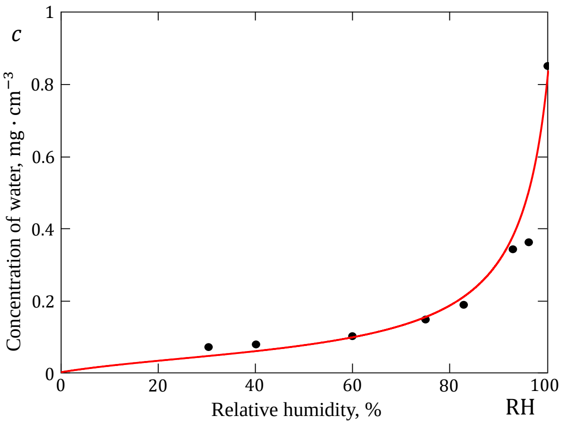

FIG. 3: Concentration of water in stratum corneum c as a function of relative humidity (adopted from [6] based on the gravimetrically measured data obtained [5] for air temperature 30°C.)

IV. DIMENSIONLESS FACTORS

The variation of the TEWL factor

$$\Upsilon_1 = \frac{1}{\bar{\lambda}} \ln\left(\frac{1-\bar{\lambda}c_0/c_{\rm h}}{1-\bar{\lambda}}\right) \tag{23}$$

is shown in Fig. 4.

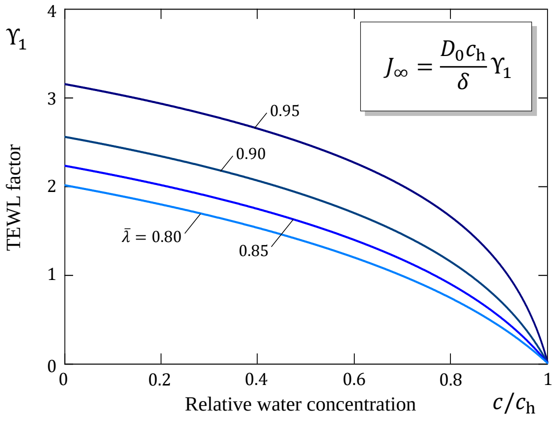

FIG. 4: TEWL factor.

The variation of the time lag factor

$$\Upsilon_2 = \frac{1}{\bar{\lambda}\Upsilon_1^3} \int_{c_0/c_{\rm h}}^{1} \frac{(1-u)}{1-\bar{\lambda}u} \ln\left(\frac{1-\bar{\lambda}u}{1-\bar{\lambda}}\right) {\rm d}u. \tag{24}$$

is shown in Fig. 5.

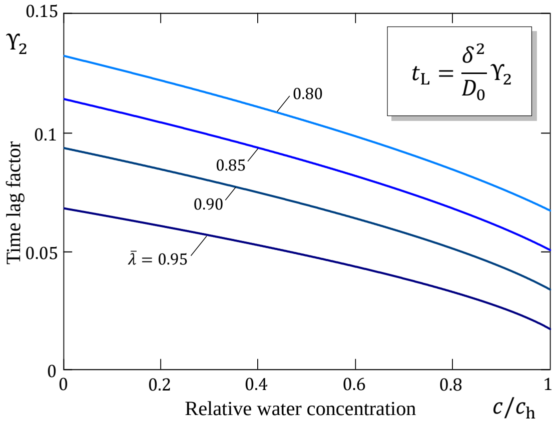

FIG. 5: Time lag factor.

The dimensionless factor Υ_3 is given by

$$\Upsilon_3 = \kappa_1 \left(1-\frac{c_0}{c_{\rm h}}\right)\left(1-\bar{\lambda}\frac{c_0}{c_{\rm h}}\right)^{-1/2}. \tag{25}$$

The other dimensionless factors are given by

$$\Upsilon_0 = \left(\frac{c_{\rm h}-c_0}{\rho_{\rm w}} + \left(1-\frac{c_{\rm h}}{\bar{\lambda}\rho_{\rm w}}\right)\ln\frac{1-\bar{\lambda}c_0/c_{\rm h}}{1-\bar{\lambda}}\right)^{-1} \ln\frac{1-\bar{\lambda}c_0/c_{\rm h}}{1-\bar{\lambda}}, \tag{26}$$

$$\Upsilon_{\rm 1d} = \Upsilon_0^{-1}\Upsilon_1, \quad \Upsilon_{\rm 2d} = \Upsilon_0^2\Upsilon_2, \tag{27}$$

$$\Upsilon_5 = \frac{1}{\bar{\lambda}}\left(\Upsilon_1 + \frac{c_0}{c_{\rm h}} - 1\right)\Upsilon_1^{-1}, \tag{28}$$

$$\Upsilon_{\rm 5d} = \Upsilon_0\Upsilon_5. \tag{29}$$

V. PARAMETRIC ANALYSIS OF THE FUJITA APPROXIMATION-BASED MODEL

We note that by expressing the wet SC thickness δ in terms of the dry SC thickness $\delta_{\rm d}$, we can evaluate the water content as follows:

$$C_{\rm w} = \delta_{\rm d}\left(\int_{c_0}^{c_{\rm h}}\left(1-\frac{u}{\rho_{\rm w}}\right)D(u)\,{\rm d}u\right)^{-1}\int_{c_0}^{c_{\rm h}} uD(u)\,{\rm d}u. \tag{30}$$

We note that the relative wet SC thickness can be represented in the form

$$\frac{\delta}{\delta_{\rm h}} = \frac{\left(1-\dfrac{c_{\rm h}}{\rho_{\rm w}}\right)\ln\dfrac{1-\lambda c_0}{1-\lambda c_{\rm h}}}{\dfrac{c_{\rm h}-c_0}{\rho_{\rm w}}+\left(1-\dfrac{1}{\lambda\rho_{\rm w}}\right)\ln\dfrac{1-\lambda c_0}{1-\lambda c_{\rm h}}}, \tag{31}$$

where $\delta_{\rm h}$ is the SC thickness in the fully hydrated state.

Finally, we note that by applying the integration by parts, we the desorption time lag can be represented in the form

$$t_{\rm lag} = \frac{(c_{\rm h}-c_0)\delta}{2J_\infty}\left(1-\frac{\displaystyle\int_{c_0}^{c_{\rm h}}\left(\int_u^{c_{\rm h}} D(w)\,{\rm d}w\right)^2{\rm d}u}{(c_{\rm h}-c_0)\left(\displaystyle\int_{c_0}^{c_{\rm h}} D(u)\,{\rm d}u\right)^2}\right). \tag{32}$$

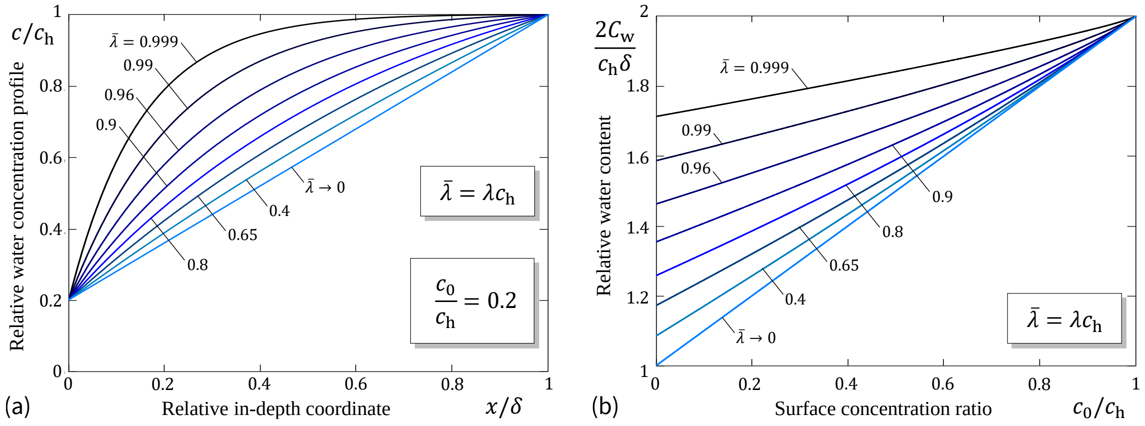

FIG. 6: Water concentration profile (a) and water content (b) in a Fujita membrane. The straight lines ($\bar\lambda = 0$) represent the constant diffusivity model.

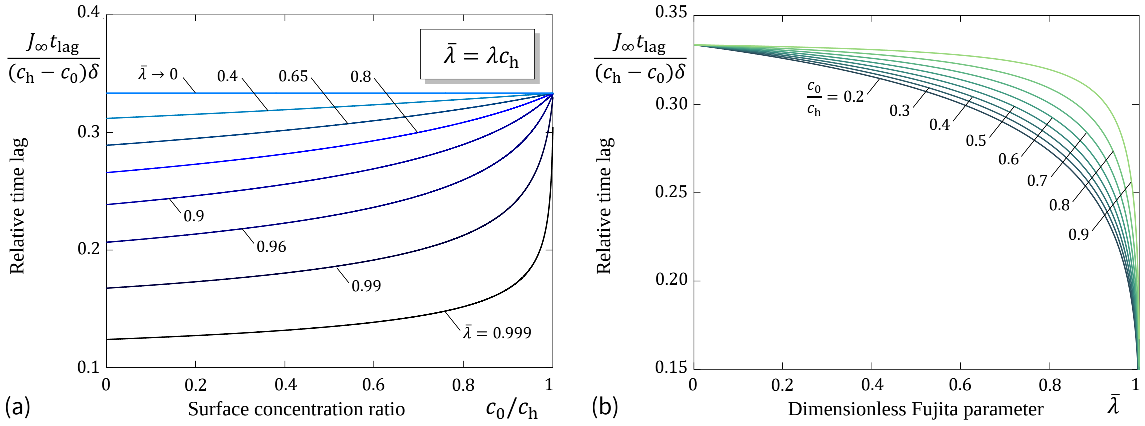

FIG. 7: The relative time lag for a Fujita membrane as a function of the surface concentration ratio (a) and the dimensionless Fujita parameter (b).

The variation of the relative SC thickness $\delta/\delta_{\rm d}$ as a function of the surface concentration ratio $c_0/c_{\rm h}$ is illustrated in Fig. 9 for the typical approximations of the SC diffusivity.

The variation of the relative membrane thickness $\delta/\delta_{\rm d}$ as a function of the surface concentration ratio $c_0/c_{\rm h}$ is illustrated in Fig. 10 for the typical surface concentration ratio $c_0/c_{\rm h} = 0.2$.

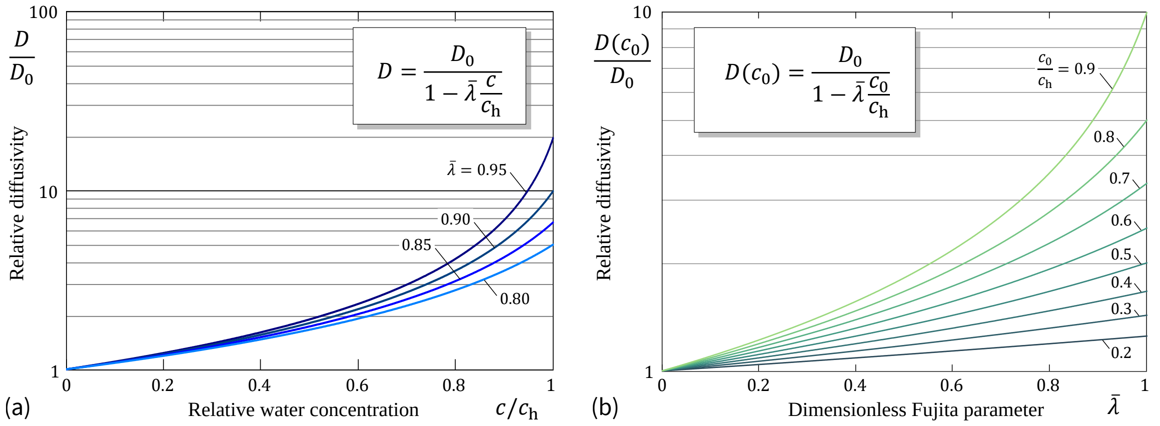

FIG. 8: a) Fujita's approximation for the SC diffusivity; b) The relative Fujita diffusivity as a function of the dimensionless Fujita parameter.

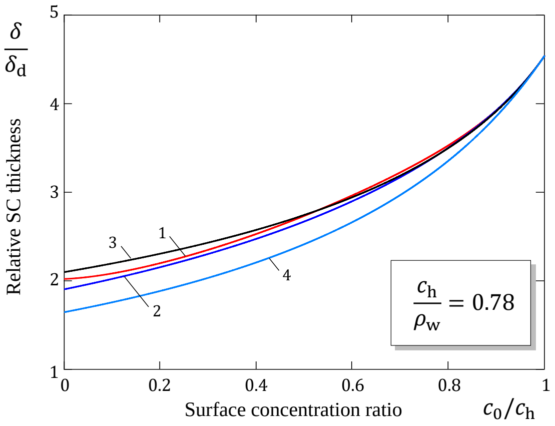

FIG. 9: The *in vivo* thickness of stratum corneum. Curves 1, 2, and 3 correspond to the biexponential approximation, Stockdale's approximation, and Fujita's approximation, respectively. Curve 4 represents the constant diffusivity model.

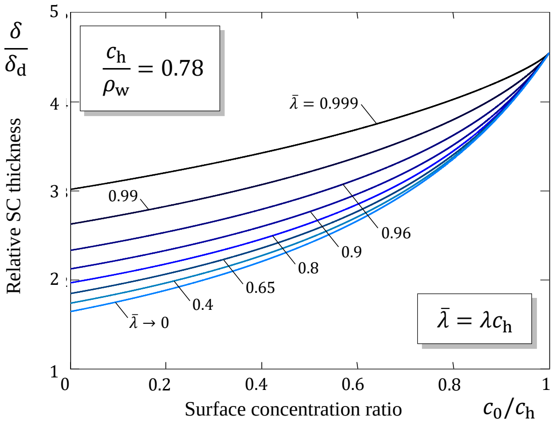

FIG. 10: The relative thickness of a Fujita membrane. The straight line ($\bar{\lambda} = 0$) represents the constant diffusivity model.

[1] J. Crank, *The Mathematics of Diffusion* (Oxford University Press, Oxford, 1979).
[2] D. M. Ruthven, Chemical Engineering Science **62**, 5745 (2007).
[3] H. Fujita, Textile Research Journal **22**, 757 (1952).
[4] D. M. Ruthven, Diffusion Fundamentals **6**, 51.1 (2007).
[5] T. S. Spencer, C. E. Linamen, W. A. Akers, and H. E. Jones, British Journal of Dermatology **93**, 159 (1975).
[6] I. H. Blank, J. Moloney III, A. G. Emslie, I. Simon, and C. Apt, Journal of Investigative Dermatology **82**, 188 (1984).
